# 

**DOI:** 10.1038/sj.bjc.6600373

**Published:** 2002-01-02


British Journal of Cancer (2002) 86(Suppl 1), S34–S121
© 2002 Cancer Research UK All rights reserved 0007–0920/02 $25.00
www.bjcancer.com

### P1

### EVALUATION OF THE VIRTUAL PATHOLOGY SLIDE: USING BREAST NEEDLE CORE BIOPSY SPECIMENS.

Sean S.P. Costello[1*], BSc; Daniel J. Johnston[1], BSc; Peter A. Dervan[2], MD, MSc, Prof; Daniel G. O' Shea[1] BSc, PhD
[1]Medical Informatics Group, School of Biotechnology Dublin City University Ireland.
[2] Department of Pathology, Mater Misericordiae Hospital, Dublin, Ireland.

The Virtual Pathology Slide is an interactive microscope emulator that presents, via the Internet, a complete digitalised tissue section of size 15.53mm x 11.61mm. VPS mimics the use of a microscope in both the stepwise increase of magnification (from 16x up to 2000x) and in lateral motion in the x and y Cartesian directions. This permits a pathologist to navigate to any area of a slide, at any magnification, similar to a conventional microscope. To learn more about the VPS access http://www.telepathology.dcu.ie.

The system incorporates a novel ability to monitor, and time a pathologist's actions while examining a VPS slide. This monitoring, coupled with online feedback forms, allows the VPS to trace the complete decision making process of pathologists, engaged in the process of microscopic diagnosis. Unique features such as a bandwidth speed test permits the examination, in real time, of user satisfaction with respect to system performance.

In order to assess, the diagnostic accuracy and acceptability of the VPS, ten wide bore breast needle core biopsies were randomly selected and presented to fifteen pathologists, with at least two years experience. The participants were required to examine each case online and provide a diagnostic classification using online feedback forms. The recorded data permitted examination of user satisfaction and inter-observer variability. Parameters measured include, time taken to complete an examination, time spent viewing a slide at a given magnification, and variability in the region of tissue examined by participants for each case.

This study evaluated the performance of the VPS as a diagnostic tool and provided an insight into the decision making process of breast needle core biopsy classification.

### P2

### SURGICAL OUTCOME OF WIRE LOCALISATION EXCISION OF MAMMOGRAPHIC LESIONS BY A PERI-AREOLAR APPROACH.

J. Ramus, C. Meyer and S. Shrotria,
Ashford and St. Peter's NHS trust, Middlesex, U.K

**Background:** Wire localisation excision is an important diagnostic and therapeutic procedure for women with benign and malignant impalpable breast lesions. The ratio of benign to malignant disease is increasing and cosmetic outcome is therefore becoming an even more important factor when planning surgery in these patients. In most practices in this country localisation excisions are done via an approach directly over the wire.

**Methods:** We studied 43 patients who had undergone wire localisation excisions of breast lesions between 1998 and 2001. All but 4 patients were operated on using a peri-areolar approach. Volumes of tissue excised, adequacy of resection for malignant disease, the need for further surgery, distance form the nipple to the lesion and subsequent cosmetic outcome were all examined.

**Results:** The average volume of tissue excised in patients with malignant disease was 142.24cm3; in patients with benign disease this was 94.35cm3. 7 patients were found to have inadequate resection margins and went on to have mastectomies. 30 patients were assessed in a special follow up clinic of which 27 were 80% or more satisfied with their cosmetic result. The distance of the lesion from the nipple did not appear to compromise adequacy of resection nor cosmetic outcome. Patients with a cytological diagnosis of malignancy pre-operatively were found to have on average 50% more tissue excised than those without.

**Conclusion:** Wire localisation excisions for impalpable breast lesions done via a peri-aereolar approach give good cosmetic results without compromising histological resection margins.

### P3

### THE VALUE OF 99mTc SESTAMIBI SCINTIMAMMOGRAPHY IN THE MANAGEMENT OF SUSPECTED LOCAL RECURRENCE IN PATIENTS WHO HAVE UNDERGONE BREAST CONSERVATION SURGERY

S. Shrotria, E.A. Bellamy, A. Chitra*
Breast Unit, Ashford Hospital, Middlesex TW15 3AA, England, UK

**Background :** The detection of local recurrence after breast conservation treatment is often difficult and frequently necessitates biopsy. 99mTc sestamibi scintimammography (SSM) is a nuclear medicine technique which provides functional breast imaging. This study was aimed to evaluate the role of SSM in the investigation and management of local recurrence.

**Patients and Methods :** Up to 130 patients were treated with breast conservation surgery annually in our breast unit. Between January 1999 and November 2001 during routine follow up 29 patients with clinical or mammographic suspicion of local recurrence were seen. SSM scans were performed in all patients and where there was suspicion of recurrence MRI scans were also carried out. In those with suspicious MRI scans a biopsy was performed for histological confirmation. Those with negative SSM result simply had long clinical follow up. Mean follow up period was 14.5 months.

**Results :** Twenty four patients had negative SSM scans. In three patients where the clinical suspicion was high, excision biopsy was carried out. Histology was benign for all three patients. The remaining twenty one patients with negative SSM scans had no evidence of clinical or radiological recurrence on long term follow up. Five patients who had suspicious SSM scans also had suspicious MRI scans, which necessitated biopsies. Histology was benign in all except one confirmed recurrence. The specificities of SSM and MRI were 85.7% and 14.2% respectively.

**Conclusion :** SSM is a useful tool in the management of patients with suspected local recurrence following breast conservation treatment. We believe that SSM should be the first line investigation in equivocal cases of suspected local recurrence. If an SSM result is negative, biopsy may be avoided in most cases and a close long term follow up may suffice. A suspicious SSM result, on the other hand is a strong indication for MRI. When both SSM and MRI are suspicious, we would recommend that histology is obtained.

### P4

### AN ANALYSIS OF BREAST CANCER PATIENTS' VIEWS ON ENTRY INTO SEVERAL CLINICAL STUDIES.

Burnet K[1], Benson J[1], Earl H[2], Thornton H[3], Cox K[4], Purushotham AD[1].
Cambridge Breast Unit[1], Department of Oncology University of Cambridge[2],
Department of Epidemiology and Public Health University of Leicester[3],
Faculty of Medicine and Health Sciences, University of Nottingham[4].

**Introduction:** Studies that have been conducted on the patient's experience of trial participation focus on such issues as informed consent, the shared decision making process and understanding trial design. Patients who have been diagnosed with breast cancer are likely to be asked to join a number of clinical studies throughout their treatment. Some research and ethics committees have expressed concerns about the number of research studies a patient may be asked to join. The opinion of patients has not been sought to support these views. The aim of this analysis was to ask the opinions of breast cancer patients about their experience of study participation.

**Patients and methods:** Ninety-six patients who had surgery for breast cancer Jan-June 2000, were sent a questionnaire on study participation. The questionnaire consisted of nine questions with space for comments. The patients were assured their results would be anonymised.

**Results:** Eighty-one out of 96 (84%) questionnaires were returned and 50 (64%) were from patients who were asked to join a clinical research study. Almost two thirds of patients who were asked to join a research study (32/50) did not believe that participation in clinical studies should be restricted (group 1). Of the remaining 18 patients (group 2) 13 stipulated the maximum number of studies that should be offered. Two of these patients quoted unrealistically high figures of 100 or 500 studies offered per patient. These patients could be included in group 1, as this does not represent a meaningful limit to trial entry. The amended figures for groups 1 and 2 are 34/50 (68%) and 11/50 (22%). Amongst the patients in group 2 who believed there should be a numerical limit on the number of studies offered, just over half, 7 (53%) considered 3 or fewer trials to be an acceptable number, whilst 2 (15%) were uncertain what the maximum number should be. Overall, 35/50 did not believe that involvement in more than one study was a significant burden. 43/50 (86%) of patients did not consider the demands of the study to be more than expected. 90% of patients felt that participation in studies was worthwhile and *no* patient regretted their decision to take part in a study.

**Conclusions:** The majority of patients believed there *should not* be a limit to the number of studies offered. This survey has further demonstrated that patients are keen to participate in research studies if they are given enough time to decide and a clear explanation in order to make an informed choice. The NHS Cancer Plan places patients' views as central to the delivery of good cancer care. Clinicians need to understand and respond to the concerns of patients who are going to increasingly be asked to enter clinical studies. This survey demonstrates that the majority of patients questioned were keen to participate in more than one clinical study, and only 3/50(16.6%) believed that the number of studies offered should be 3 or less. On the other hand 90% of patients felt that participation in studies was worthwhile and *no* patient regretted her decision to take part in a clinical research study.

### P5
### MODULATION OF CDC2 KINASE-SURVIVIN EXPRESSION IN BREAST CANCER APOPTOSIS

[#] SH Teh*, ADK Hill, A Lee, L Young, E McDermott, N O'Higgins. Depart. of Surgery, St Vincent's University Hospital, Ireland. Depart. of Surgery[#], Mayo Clinic, USA

**Aims:-**Mechanisms by which basic fibroblast growth factor (bFGF) interacts with COX inhibitors to dysregulate breast cancer cell apoptosis were investigated. **Methods and Results:-** Stimulation of MCF-7 cancer cells with (bFGF) decreased apoptosis and induced up-regulation of the mitochondrial protein, Bcl-2 without altering the expression of Bax. COX inhibitors, meloxicam (selective COX II) and aspirin (non-selective) but not SC 560 (selective COX I) induced cancer cell apoptosis as assessed by quantifying total intracellular nucleosomes and confirmed by TUNEL. Pre-treatment with meloxicam and aspirin modulated the anti-apoptotic effect of bFGF. bFGF stimulation rapidly increased survivin and phospho-cdc2 expression. COX inhibitors decreased basal expression of Bcl-2, survivin and prevented their up-regulation seen with bFGF pre-treatment. Phosphorylation of cdc2 by bFGF was reduced in the presence of meloxicam and aspirin. COX II expression however was low and not inducible by bFGF. $PGE_2$ was unaltered in both control and bFGF groups. **Conclusion:-**These findings suggesting that COX inhibitors modulate breast cancer cell survival by interfering both cell cycle-independent (mitochondrial Bcl-2) and a cell cycle-dependent (cdc2 kinase-survivin) apoptosis pathways independent of COX II activities. The anti-survivin properties of aspirin and meloxicam in our i*n vitro* model may explain the chemoprevention role of COX inhibitors.

### P6
### SENTINEL NODE BIOPSY IN BREAST CANCER: A COMPLETE IMMUNOHISTOCHEMICAL STUDY.

N.Beechey-Newman *[1], H.Hamed[1], J.Skillman[1], C.d'Ariggo[2], S.Clark[3], I.S.Fentiman[1].
1.Breast Unit, Guy's Hospital, London SE1 9RT.UK
2.ICRF Labs, Guys Hospital, London SE1 9RT. UK.
3.Dept of Nuclear Medicine, Guys Hospital, London SE1 9RT. UK.

**Aims.** To discover the true accuracy of sentinel node biopsy (SNB) by eliminating thev bias that results from the more intensive histological assessment of the SN than non-SNs.

**Methods.** Three hundred unselected (T1-3, N0-1), consecutive patients with primary breast cancer had sentinel biopsy followed by level III axillary clearance. SN localisation was by isotope (0.1ml nanocol, intra-dermal, lymphoscintigram) and dye (0.5 – 1.0ml patent blue V, intra-dermal) in 112 patients and dye only in 188 patients, the distribution being dependent on the availability of nuclear medicine facilities on the day of surgery and the consent of the patients. All sentinel and non-sentinel nodes were treated identically by serial sectioning followed by H&E and immuno-histochmistry for cytokeratin (CAM 5.2).

**Results.** A SN was identified in 250 (83.3%), 91.9% for isotope and dye compared to dye alone, 78.2%, p=0.005). A mean of 1.4 SNs and 21.8 non-SNs were retrieved. The SN predicted the status of the rest of the axilla with an accuracy of 91.2% and a sensitivity of 82.3%. There was no difference in the sensitivity of SNB between the methods of localisation used (isotope and dye 84.6%, dye alone 81.1%). Statistical analysis including logistic regression of 10 patient and methodological variants (age, tumour site, tumour size, histological type and grade, high node positivity, dye volume, previous excision biopsy, needle localisation and surgeon) demonstrated that lobular histology (p=<0.05)and high node positivity (≥ 4 positive nodes) (p=0.006) were independent factors associated with reduced SN identification. Needle localisation (p=0.062) and retroareolar tumour (p=0.058) site were close to being significant. None of the same 10 biological and methodological factors affected SNB accuracy.

**Conclusions.** In this prospective study when patients are not selected and all of the axillary nodes are cleared and treated by similar histological techniques the accuracy and sensitivity of SNB is not as accurate as generally quoted. Different biological and methodological techniques affect the rate of identification of the SN but not its accuracy.

### P7
### BREAST DUCT MICRO-ENDOSCOPY: A STUDY INTO THE FESIBILITY AND TECHNIQUE

N. Beechey-Newman* FRCS[1], A. Kothari MS[1], H. Hamed FRCS[1], IS. Fentiman FRCS [1..]
1.Breast Unit, Guy's Hospital, London SE1 9RT .UK.

**Aims**. To assess the feasibility and technical factors that influence the successful visualisation of mammary duct epithelium using a purpose built breast micro-endoscope.

**Methods** We conducted ex-vivo duct micro-endoscopy on 35 mastectomy specimens. Visualisation of mammary duct epithelium was achieved using a solid rod Depth of Field Imaging (DOFI[R] Mini) micro-endoscope consisting of a 0.89mm maximum outer diameter rigid sheath, optical fibre and a working channel of 0.35mm. Insufflation was with air and saline.

**Results** We could successfully visualise the duct epithelium in all 35 specimens, including in 2 specimens with inverted nipples. We examined 104 ducts in all; an average of 3.3 (median 3) ducts per specimen. Visualisation of the proximal ducts (first 2 cm) only was possible in 12/35 (34%). Navigation beyond 2 cm was possible in 23/35 or 66%. In 18/104 ducts we were able to navigate beyond a depth of 5 cm. The maximum depth we were able to navigate to was 9.2 cm. In ducts with a pathological discharge distal navigation was achieved in 100% of the cases. Visualisation beyond three bifurcations of the ductal system was achieved in 32% of ducts. We could visualise pathological changes to duct epithelium in 8/35 patients who had DCIS or invasive malignancy.

**Conclusion** Breast duct micro-endoscopy is technically feasible. Besides having a role in patients with pathological nipple discharge, current techniques enable the examination of multiple normal calibre ducts. With improved instrumentation this procedure could have use in the surveillance and diagnosis of breast cancer.

### P8
### PAGET'S DISEASE OF THE NIPPLE: A MULTI-FOCAL MANIFESTATION OF HIGHER RISK DISEASE.

Ashutosh Kothari [1] MS*, Nicolas Beechey-Newman [1] FRCS, Hisham Hamed [1], FRCS. Corrado D'Arrigo [2], MRCPath. Andrew M. Hanby[3], FRCPath. Ken Ryder [2] C.Stat. Ian S. Fentiman FRCS [1]
1. Breast Unit, Guy's Hospital, London SE1 9RT. United Kingdom.
2. ICRF Laboratories, , Guy's Hospital, London SE1 9RT. United Kingdom.
3. Department Of Histopathology, St. James' University Hospital. Leeds.

**Background.** The treatment of Paget's disease by mastectomy has recently been challenged in favour of breast conserving techniques. A large series of patients treated with mastectomy has been reviewed to assess the feasibility of less radical surgery.

© 2002 Cancer Research UK

**Methods.** 70 women with a clinical diagnosis of Paget's disease were reviewed. The type, grade, receptor and node status, and the mammographic and pathological extent of the underlying breast malignancy were determined. The survival of patients with invasive disease was compared with matched controls without Paget's disease.

**Results.** The underlying malignancy was invasive in 58%. Despite the fact that only one third presented with a palpable mass the malignancy was frequently extensive, being confined to the retro-areolar region in only 25%. The true extent of the disease was underestimated by mammography in 43% of cases. Of the patients with DCIS 96.5% were high grade and 100% of the invasive cancers were also of high cyto-nuclear grade. Over-expression of the c-erb-B2 oncogene was detectable in 83%. Patients with Paget's disease had a significantly worse survival than matched controls, but this difference was eliminated if they were also matched for c-erb-B2 status.

**Conclusions.** Paget's disease is often associated with extensive underlying malignancy which is difficult to accurately assess either clinically or mammographically. As a consequence cone excision of the nipple would have resulted in incomplete excision in 75% of cases. In Paget's disease the underlying malignancy is of high grade and is frequently c-erb-B2 positive with a resulting poor prognosis. Aggressive local and systemic treatment therefore would seem to be merited.

### P9
### INTERACTIONS BETWEEN ALPHA-1-ACID GLYCOPROTEIN AND TAMOXIFEN AND ITS METABOLITES MAY AFFECT DRUG EFFICACY

SC Paterson *[1] and KD Smith [1]
[1] Department of Bioscience, University of Strathclyde, Glasgow, G1 1XW

BACKGROUND: Several plasma proteins, of which alpha-1-acid glycoprotein (AGP) is one, are known to reversibly bind to drugs. If a drug is bound to plasma components such as albumin or AGP, it may not be pharmacologically active, due to alterations in distribution and clearance, and this is of consideration in the design of drug dosage schedules. In addition, the concentration of AGP has been shown to increase two- to five-fold (a process termed the acute phase response) in disease states, such as cancer, resulting in potentially higher binding of drug to AGP. Thus, the binding of tamoxifen, a commonly used drug in the treatment of breast cancer, to AGP was investigated. METHODS: The technique employed was a fluorescence quenching method, which has the benefits of being quick, sensitive and accurate. It can be performed in a cuvette, or a 96-well microtitre plate if the quantity of drug or protein is small. The method is based on the fluorescence of tryptophan residues, present in the AGP molecule, at 280nm excitation. Upon binding of the drug to AGP, the level of fluorescence is decreased, whereas if no binding occurs, there is no decrease in fluorescence. The drugs investigated were tamoxifen citrate, tamoxifen free base and the physiologically active metabolite 4-hydroxytamoxifen. RESULTS: When the fluorescence of 0.83mg/ml AGP at 300-400nm emission was recorded, a decrease in peak fluorescence at 335nm of 34% was noted after addition of 1μM tamoxifen free base to the AGP solution, indicating that the drug was partially bound to AGP. Addition of 5μM and 10μM tamoxifen free base resulted in decreases in fluorescence of 87% and 96% respectively, which suggests that very little unbound drug would remain in the plasma. At concentrations above 10μM, quenching was greater than 98%. Tamoxifen citrate was found to bind AGP to a lesser extent than that of the free base, with decreases in fluorescence of 23%, 72% and 91% following the addition of 1μM, 5μM and 10μM drug, respectively. Quenching of 99% was observed after addition of 50μM tamoxifen citrate, suggesting almost complete binding of drug to AGP at this concentration. The presence of 4-hydroxytamoxifen, at 25μM, resulted in a decrease in fluorescence of 55% compared to AGP solution alone. This was much lower than the binding observed for either tamoxifen free base or tamoxifen citrate. In stark contrast to tamoxifen free base, 96% decrease in fluorescence was found for 4-hydroxytamoxifen at 250μM, while the same fluorescence decrease was seen with only 10μM tamoxifen free base. CONCLUSION: In summary, the results indicated that tamoxifen free base binds to AGP slightly more than does tamoxifen citrate, however 4-hydroxytamoxifen is bound much less by AGP. Since tamoxifen must be metabolised by the body to 4-hydroxytamoxifen, binding of the pro-drug to AGP may hamper metabolism, hence it may take longer to achieve pharmacologically therapeutic levels of 4-hydroxytamoxifen in vivo, thus possibly reducing the efficacy of the drug.

### P10
### COREGULATORS AND ESTROGEN RECEPTOR FUNCTION IN THE BREAST

FJ Fleming*, ADK Hill, M J Duffy, EW Mc Dermott, N O'Higgins, L Young
Department of Surgery, University College Dublin, Dublin, Ireland

The estrogen receptors, ER-α and ER-β, function as transcription factors to modulate expression of target genes. ER-α interacts with 'coregulators' to enhance or inhibit transcription. We hypothesised that these coregulators contribute to the differing clinical response of patients to treatment with tamoxifen. The aim of this project was to localise ER-α and ER–β, the coregulator SRC-1 and the corepressor SMRT within breast tissue and to examine the ability of tamoxifen and β-estradiol to regulate the expression of ER-α and SRC-1.

ER-α, ER–β, SRC-1 and SMRT were localised within paraffin embedded human breast tissue (n=11) by immunohistochemistry. Strong positive staining for ER-α (10/11) and ER-β (2/11) was detected in the nuclei of invasive ductal carcinoma and invasive lobular carcinoma. SRC-1 (2/11) and SMRT (1/11) were found to be expressed to a lesser extent and were localised to nuclei of tumour cells. The ability of four hydroxytamoxifen (4-HOT) and β-estradiol to regulate the protein expression of ER-α and SRC-1 in the breast tumour cell line MCF-7 was determined using Western Blotting. ER-α was expressed within the cytoplasm and to a greater extent the nucleus of MCF-7 cells. Both 4-HOT and β-estradiol induced an upregulation of nuclear ER-α. ER-α translocated from the cytoplasm to the nucleus in the presence of β-estradiol. SRC-1 was located within the nuclear fraction of MCF-7 cells and was up regulated in the presence of β-estradiol.

Localisation of the coregulator SRC-1 within human breast tissue and the regulation of SRC-1 by β-estradiol in the breast tumour cell line MCF-7 is new evidence that coregulators modulate ER activation by β-estradiol and tamoxifen.

### P11
### DIETARY PHYTOESTROGENS INHIBIT STEROID METABOLISM AND MAY INFLUENCE THE EXPOSURE OF BREAST CANCER CELLS TO ENDOGENOUS ESTROGENS.

D Rea[1]*, R M Harris[2], D M Wood[2], P J Hughes[3], R H Waring[2], & C J Kirk[2],
[1] Institute for Cancer Studies, [2] School of Biosciences, [3] Division of Immunology, University of Birmingham, B15 2TT

Environmental estrogens are a diverse group of compounds that can exert estrogenic effects in living organisms. They include the "phytoestrogens", such as genistein and daidzein, which are natural constituents of soy-rich diets and which have been suggested to confer a reduced risk of developing breast cancer.

Although some effects of environmental estrogens may be mediated by estrogen receptors, there is growing evidence that these compounds can influence endocrine balance by acting as substrates or inhibitors of key steroid-metabolising enzymes. We have investigated the influence of dietary phytoestrogens upon the activities of sulphotransferase and sulphatase enzymes active against estrogens in human platelet preparations. These are SULT1A1, the enzyme thought to be responsible for sulphating estrogens in MCF7 cells and in human breast tumours, and steroid sulphatase.

Genistein, daidzein and other, less abundant dietary phytoestrogens were found to be inhibitors of both enzymes in human platelet preparations. (see table).

| Phytoestrogen | $IC_{50}$ against: | |
|---|---|---|
| | Steroid sulphatase vs. 1μM estrone sulphate | SULT1A1 vs. estradiol (10nM) |
| Genistein | >25μM | 0.8μM |
| Daidzein | >50μM | 1.0μM |
| Quercetin | n.d. | 0.5μM |
| Luteolin | >25μM | 0.4μM |

© 2002 Cancer Research UK

SULT1A1 is very much more sensitive to inhibition by dietary phytoestrogens than is steroid sulphatase. In the presence of circulating concentrations of genistein and daidzein reported to exist in individuals consuming a high soy diet (~1μM), SULT1A1 activity would be significantly inhibited whilst that of steroid sulphatase would be unaffected. This combination of effects upon the balance of estrogen sulphation/de-sulphation within breast tissue might be predicted to increase exposure to active (de-sulphated) estrogens. However, in postmenopausal women, a significant pool of potentially active estrogen circulates as estrone sulphate synthesised from adrenal-derived DHEA, via the action of sulphotransferases and other steroid metabolising enzymes. Hence inhibition of sulphotransferase activity by phytoestrogens in postmenopausal women may reduce this pool of circulating estrone sulphate thereby reducing the exposure of tissues to active estrogens.

**P12**

**MUTATIONAL ANAYSIS OF P53 OF PRIMARY BREAST CANCER FROM FINE NEEDLE ASPIRATION; CORRELATION WITH CLINICAL OUTCOME AFTER NEOADJUVANT CHEMOTHERAPY**

Marina Parton[1]Gillian Howes[2*]Ian Smith[1]Steve Humphreys[2]Mitch Dowsett[1]
Academic Department of Biochemistry, Breast Unit, Royal Marsden NHS Trust, London SW3 6JJ, UK; [2]The Marilyne Welsh Research Unit, Cellular Pathology Department, Preston Hall Hospital, Maidstone, Kent, ME20 7NH, UK *Presenting author

**AIM**: To assess the impact of mutations in the p53 gene on clinical response of breast carcinomas to chemotherapy. **METHOD:** Patients undergoing primary (neo-adjuvant) chemotherapy for breast cancer between April 1994 and October 1998 were asked to participate in a fine needle aspiration (FNA) study of breast tumours. FNAs were taken prior to treatment using a 23G needle and cytospins were prepared and air-dried before storage at -80°C. DNA was extracted from the cells using the Puregene Kit (Flowgen Ltd, UK) according to their standard protocol. Frozen MCF-7 cells were processed in parallel as controls. The p53 gene (exons 5-8) was amplified using a nested PCR regime, and sequenced manually. All mutations were confirmed on the opposite strand by further sequencing. The effect of a mutation on clinical response to chemotherapy was tested for significance using the Mann Whitney test with trend. Survival & progression free survival were determined by the life-table method of Kaplan & Meier and comparisons were made by means of the logrank test. **RESULTS:** Forty-six of 53 available samples examined were successfully analysed. A total of 14 alterations in 13 tumours (30%) were found in exons 5 – 8 of the p53 gene, 11 predicted functional modification of the protein. Of the mutations found, there were 7 base substitutions (51%), 1 deletion (7%), 2 silent mutations (14%), 2 mutations affecting gene splicing (14%) and 2 non-sense mutations (14%). Six mutations (43%) were present in, or disrupted the L2 and/or the L3 region of the gene, which are
involved in DNA binding. Thirty-six patients out of 44 (82%) assessable for response to chemotherapy had a clinical response (6 no change, 2 progressive disease). Two patients could not be evaluated; both patients were found to have WTp53. No association was found between presence of p53 mutation (including L2/L3 region mutations only) and response to chemotherapy. Median relapse free survival (RFS) at 3 years was 74% overall, but was 64% for those with a mutation and 78% for those without (n.s). When the mutational data was spilt into two groups according to whether the mutation was in the L2/3 regions, RFS at 3 years was 50% for those with a L2/3 affected mutation and 80% for those with mutations in other areas of p53 (n.s).**CONCLUSIONS:** This analysis confirms the use of this method of DNA sequencing in FNAs as suitable for breast cancers. The incidence of mutation is consistent with that in other publications. Mutational states did not predict response, but showed a trend to an association with RFS.

**P13**

**A RETROSPECTIVE AUDIT OF THE MANAGEMENT OF LEPTOMENINGEAL CARCINOMATOSIS FROM BREAST CANCER**

DP Jackson[1], C Siller*[1], C Sriwardhana[1], TJ Perren[1].
[1]Cancer Research UK Clinical Centre, Division of Cancer Medicine Research, St. James's University Hospital, Beckett Street, Leeds, LS9 7TF

Leptomeningeal carcinomatosis (LC) is a common manifestation of metastatic disease in solid tumours, occurring in 4-15% of patients. Breast cancer is the primary tumour most commonly associated with this condition. We have conducted a retrospective audit of the management of LC from breast cancer, in the Professorial Medical Oncology Unit in Leeds, during the period from January 1994 to March 2001.

17 patients diagnosed as having LC secondary to breast cancer were identified during this period, 15 of whom have subsequently died. The incidence of LC was 5.8%. The mean age at diagnosis was 52 (range 35-70 years).

The women usually presented with multiple neurological symptoms, including headache, cranial nerve palsies, fitting and vomiting. 14 (82%) underwent investigation with lumbar puncture (LP), 11 (65%) had CT, 7 (41%) had MRI and 2 (12%) had EEG. Only 50% of LPs yielded cytologically malignant cells, although an additional 41% showed other abnormalities such as atypical cells or raised protein. CT scan produced a diagnostic or suspicious result in only 36%, while MRI was diagnostic or suspicious in 85% of the patients. Diagnosis was made on a combination of clinical assessment, CSF analysis and radiological imaging. Thirteen of the women (77%) had 2 or more additional sites of metastatic disease, while in 2 women (12%) LC was the only site of metastasis.

Of the 17 women, 4 received no treatment either because they refused or were considered too unwell. 12 patients received intrathecal (IT) chemotherapy. 1 received radiotherapy alone, while 2 patients received radiotherapy as part of multi-modality therapy. 7 of the patients who were given IT treatment also received systemic chemotherapy. Response to treatment was determined by clinical improvement, disappearance of malignant cells from the CSF or resolution of leptomeningeal changes on MRI. 5 of the 12 patients (42%) receiving treatment including IT chemotherapy showed evidence of response to treatment.

The median survival following diagnosis of LC was 17 weeks for all 17 patients with LC, and 20 weeks for the 12 patients who received treatment including IT chemotherapy. However there were some longer term survivors: 2 patients survived for 3 years 10 months and 2 years 1 month, while a further 2 patients remain alive at 14 months and 6 months.

Data from this audit & a review of the literature has been used to compile proposed guidelines for the management of LC within the Yorkshire Cancer Network.

**P14**

**MAMMAGLOBIN A: A BREAST SPECIFIC MARKER.**

*N. A. O'Brien[1], T. M. Maguire[2], N. O'Donovan[3], A. Hill[2], E. McDermott[2], N. O'Higgins[2] and M J. Duffy[1].
[1]Departments of Nuclear Medicine, [2]Surgery and [3]Medical Oncology, St. Vincent's University Hospital, Dublin 4,

Mammaglobin A (MGA) is a recently identified gene which appears to be expressed specifically in breast tissue. MGA is thus a potential marker for breast cancer. In this investigation, expression of MGA and mammaglobin B (MGB) were measured at the mRNA level by RT-PCR in 142 invasive breast carcinomas, 32 fibroadenomas and 21 specimens of normal breast tissue. MGA was detected in 86/142 (61%) of the breast cancers. In the cancers, expression was found more frequently in estrogen receptor-positive than estrogen receptor-negative samples ($p < 0.01$). Expression however, showed no significant relationship with either tumor size, axillary node status or progesterone receptor status. mRNA for MGB was present in 78/142 (55%) of breast cancers. MGB was found more frequently in cancers positive for MGA than in samples negative for this mRNA ($p < 0.001$). Overall, 73% of the cancers expressed either MGA or MGB. MGA was detected in only 1/23 normal non-breast tissues and in none of 10 non-breast cancers. MGB was present in 4/14 non-breast normal tissues and in 1/8 non-breast malignancies. We conclude that expression of MGA is almost specific for breast tissue. MGA may therefore become the first relatively breast-specific marker. MGB, on the other hand, is less breast-specific but its presence may complement MGA in the detection of breast cancer.

© 2002 Cancer Research UK

### P15
### A PHASE II TRIAL USING ETANERCEPT, A RECOMBINANT TUMOUR NECROSIS FACTOR ANTAGONIST, IN METASTATIC BREAST CANCER

Forster M. D.*, Braybrooke J., Madhusudan S., Kaur K., Hoare S.°, Balkwill F.°, Ganesan T. S., Talbot D., Harris A. L.
Cancer Research UK Medical Oncology Unit, Churchill Hospital, Oxford OX3 7LJ, U.K.,
°Cancer Research UK Translational Oncology Laboratory, St. Bartholomew's Hospital, London, EC1M 6BQ, U.K.

Purpose: There is preclinical data to support the role of TNF in the pathogenesis of breast cancer. This activity is partially via angiogenesis and is mediated through activating secondary cytokines including MCP-1 and IL-6. Preclinical studies have shown Etanercept to effectively decrease the biological activity of TNF. This phase II trial analysed the antitumour and biological effects of Etanercept in patients with metastatic breast cancer.
Methods: Etanercept was given at a dose of 25mg SC twice weekly. The treatment was planned for a minimum of 3 months and a maximum of 1 year with objective re-evaluation every 3 months. Surrogate markers used to measure the biological effects of TNF were plasma MCP-1 and a novel whole blood cytokine release assay of IL-6 and MCP-1. These were measured pre-treatment, at 24 hours, 7 days, 28 days and 4 weekly there after.
Results: 12 female patients with ductal or lobular breast cancer enrolled had a median age of 54 (34-74 yrs) and performance status ≤ 2. All patients had metastatic disease and had had multiple previous lines of therapy. There were no significant toxicities. All patients progressed despite therapy, with 4/12 receiving at least 3 months treatment. Plasma MCP-1 was essentially unchanged with a mean rise of 296pg/ml to 454pg/ml. There was a gradual decrease in the MCP-1 release assay which was most pronounced at day 84 (46% of pre-treatment values). There was also a decrease in the IL-6 release assay at day 1 which was sustained at day 84 (27% of pre-treatment values).
Conclusions: Etanercept has biological effects in patients with breast cancer, which are being evaluated further. The trial is ongoing with a dose increase to 25mgs thrice weekly. Further studies are warranted in combination with other antiangiogenic agents that inhibit different pathways.

### P16
### CELL LINE SPECIFIC RESPONSE OF THE P53 PATHWAY TO TAMOXIFEN IN BREAST CANCER

A Ingram[1], D Ziyaie*[1], KL Ball[1], TR Hupp[2], AM Thompson[1]
[1]Dept. of Surgery & Molecular Oncology, [2]Dept. of Molecular & Cellular Pathology, Ninewells Hospital & Medical School, Dundee DD1 9SY.

**Background:** The anti-oestrogen tamoxifen remains the first-line endocrine treatment of choice for women with tumours positive for oestrogen receptors. Tamoxifen's anti-oestrogenic activity is in part attributed to its high affinity for binding to oestrogen receptors with subsequent alteration in the transcriptional activity normally attributed to the oestrodiol-bound oestrogen receptor complex. However not all patients with receptor positive tumours respond to the primary tamoxifen treatment. The exact mechanism by which tamoxifen inhibits tumour growth or, molecular changes that lead to tamoxifen resistance are not known.
**Aims:** To examine the relation of the anti-tumour properties of tamoxifen with p53 and the p53-transactivated target gene p21 in breast cancer cell lines which differ in their p53 and oestrogen receptor status.
**Methods:** Protein and mRNA expressions in response to tamoxifen treatment were determined by western and northern blot analyses respectively using highly sensitive human specific p53 monoclonal antibodies in $ER^+$/ $p53^{active}$, $ER^+$/$p53^{mutant}$, $ER^-$/$p53^{mutant}$, $ER^+$/$p53^{dominant\ negative}$ breast cancer cell lines.
**Results:** In response to tamoxifen treatment a transient induction of p21 at the protein level in the absence of p53 protein induction was observed in MCF-7 oestrogen receptor positive, wild-type (active) p53, tamoxifen sensitive breast cancer cell lines. However p53 protein in tamoxifen treated cells was not phosphorylated at Ser392, a site whose modification is a sensitive probe for p53 activation in the absence of changes in p53 protein levels. This phenomenon was not observed in any of the other cell lines.
**Conclusion:** These data suggest that p21 protein induction may be independent of p53 protein activation in $ER^+$/ $p53^{active}$ breast cancer highlighting an alternative pathway for tamoxifen action.

### P17
### CD55 AND CD59 AS INDEPENDENT INDICATOR OF POOR PROGNOSIS IN BREAST CANCER PATIENTS

Madjd Z*✞, Durrant LG✞, Carmichael J✞, Pinder S✠, Ellis I✠
✞CRC Academic Unit of Clinical Oncology, ✠Division of Histopathology , University of Nottingham, City Hospital, Hucknall Road, Nottingham, NG5 1PB

A better understanding of the molecular recognition and stimulation of immune responses has led to the development of many new approaches for the immunotherapy of cancer. CD59 (protectin) and CD55 (DAF) are membrane-associated complement regulatory proteins which inhibit complement–mediated lysis of autologous cells. CD59, a phosphatidyl-inositol-anchored glycoprotein, inhibits the formation of terminal membrane attack complex (MAC) of complement and is a second ligand for CD2 contributing to T-cell activation. CD55, otherwise known as DAF (decay accelerating factor), is a GPI-anchored protein composed of 4 contiguous short consensus repeat (SCR) domains and a Serin/Threonine (S/T) rich region proximal to the cell surface which inhibits the complement cascade by inactivating C3 convertases and preventing C3b deposition on cell membranes. These complement proteins have been shown to be over-expressed by a range of solid tumours and have been implicated in the limited success of antibody-mediated lysis of tumours. The levels of expression of these complement regulatory proteins may correlated with breast cancer progression and will aid in the design of new cancer vaccines. Over-expression of CD55 has been shown to be an independent indicator of poor prognosis in colorectal cancer patients.
**Aim:** The aim of present study is to determine whether expression of these antigens on breast tumours is associated with patient prognosis. There are multiple prognostic factors in breast cancer including size, lymph node, stage, differentiation and proliferation. The NPI is an integrated prognostic index based on tumour size, LN stage, and tumour grade, which used to predict patient survival for women with invasive breast cancer.
**Material and method:** In this study the reactivity of anti- CD55 (clone 67) and anti-CD59 (MEM-43) on 300 and 600 breast carcinomas has been investigated by immunohistochemical staining using tissue microarrays. The level of expression of these antigens on breast tumours and tumour prognosis (grade, stage, size, oestrogen receptor, and NPI) has been evaluated.
**Results:** Most breast carcinomas express CD55 weakly, however, CD59 was strongly expressed on 20% of breast tumours. Good prognosis patients (NPI1) had lower expression of CD55, whereas poor prognosis patients (NPI3) expressed higher levels of CD55 (P=0.016). CD59 expression was correlated with the grade of differentiation i.e., higher expression of CD59 was more often found in well –and moderately differentiated tumours and lower levels of CD59 was found in poorly differentiated (P<0.001). Significant correlation was found between the percentages of cells stained for CD59 and survival (P<0.005). In contrast, no correlation was found between the percentage of cells expressing CD59 and multiple prognostic factors apart from tumour grade, indicating this may be an independent prognostic indicator for breast carcinomas.

### P18
### INTRAOPERATIVE DETECTION OF METASTATIC AXILLARY NODES IN EARLY BREAST CANCER

Salem AA*, Douglas-Jones AG and Mansel RE, University of Wales College of Medicine, Cardiff,

**Background:** Sentinel Node Biopsy (SNB) proved to be a reliable predictor of the axillary node status, which could help in operating on node-positive patients only. However, in order to avoid a second operation, a fast and reliable method for the intraoperative evaluation of the sentinel node(s) is needed.
**Aim**: is to develop a fast and reliable method for the pathological evaluation of the sentinel nodes intraoperatively.
**Methods**: We developed a protocol for rapid Immunohistochemistry (IHC) staining of touch imprints from axillary nodes intraoperatively and tested it on 349 nodes from 32 patients with early breast carcinoma who underwent Axillary Node Clearance (ANC) or SNB, and compared the results with those of permanent Haematoxylin and Eosin (H&E) sections. IHC-stained paraffin sections were done whenever the results of IHC-stained imprints were different from those of the H&E sections.

© 2002 Cancer Research UK

**Results**: 30 nodes from 10 patients were positive on IHC and 29 nodes from 9 patients were positive on H&E. Two nodes were positive on IHC imprints and negative on H&E sections and that was confirmed on IHC sections. One of these two nodes was the only sentinel node in one patient. Only one of two positive nodes in an ANC was missed by IHC during the learning curve. On a patient basis, sensitivity was 100% and 90%, and Negative Predictive Value (NPV) was 100% and 96% for IHC and H&E respectively. On a node basis, sensitivity was 97% and 93%, and NPV was 99.6% and 99.3% for IHC and H&E respectively. Because of the absence of false positives, specificity and Positive Predictive Value (PPV) were 100% for both IHC and H&E.
**Conclusion**: IHC-stained touch imprints could provide a sensitive and fast method for the intraoperative evaluation of axillary lymph nodes in breast cancer patients.

**P19**

**NEO-ADJUVANT CHEMOTHERAPY HAS NO EFFECT ON MAPK AND p38 ACTIVATION IN BREAST CANCER PATIENTS**

AA Liem*[1], MVCL Appleyard[1], MA O'Neill[1], TR Hupp[2], WSR Hew[2], MP Chamberlain[3], CR Wolf[3] and AM Thompson[1], [1]Dept. of surgery & Molecular Oncology,[2]Dept. of Molecular & Cellular Pathology,[3]Biomedical Research Centre, University of Dundee, DD1 9SY,U.K.

**Introduction** Mitogen-activated protein kinases (MAPK) and p38 are involved in coordinating cellular responses to stress, with alterations in these pathways being involved in cancer pathogenesis. There is evidence of constitutive MAPK activation and expression in human breast cancer tissues. p38 activity in human tissues is not well documented. The aim of this study was to examine MAPK and p38 activity in human breast cancer tissues treated with combination chemotherapy containing doxorubicin and compare this with cancers not exposed to chemotherapy.
**Materials and Methods** Human breast cancer tissues (0.5 $cm^3$) were obtained at mastectomy in compliance with MRC guidelines from 18 patients who underwent neo-adjuvant chemotherapy and 14 previously untreated patients. Following tissue lysis, supernatants were recovered to determine kinase activity. MAPK and p38 activity was determined by kinase assay for the appropriate substrate and western blot analysis. MAPK and p38 activities in the neo-adjuvant and untreated groups were correlated with the Nottingham Prognostic Index (NPI). As doxorubicin is known to act on the mechanisms involved in the multidrug resistance phenomenon, Pgp expression in 10 neo-adjuvantly treated tissues was also determined via immunoblotting and immunohistochemistry.
**Results** MAPK and p38 activity could be shown in both groups of patients. Similar patterns of MAPK and p38 activity were seen in individual patients, but were unrelated to whether the patient had previous neo-adjuvant chemotherapy. No correlation was observed between either kinase activities and the NPI. Pgp expression was not demonstrated in 5 of the neo-adjuvantly treated cancers.
**Conclusion** The similar patterns found for each patient with respect to MAPK and p38 activity suggests their interaction within a given tumour rather than any association with the use of doxorubcin. Furthermore, the lack of correlation found between alterations in kinase activity, known to play a role in drug resistance, and Pgp expression, could suggest the importance of timing between drug exposure and mastectomy, where early mastectomy following neo-adjuvant treatment might prevent Pgp expression and therefore drug resistance.

**P20**

**BLACK WOMEN WITH BREAST CANCER ARE MORE LIKELY TO BE ER-BETA POSITIVE**

*Keith A. Dookeran , Jie Yang, Marin Sekosan[1], Elona Sciupokiene, Wendy Rogowski, Hung Tran, Rose Catchatourian[2], & Howard A. Zaren.
Departments of Surgery & Pathology[1], and Division of Medical Oncology[2], Cook County Hospital, 1835 West Harrison Street, Chicago, IL 60612 USA.

**Introduction:** A recent study has demonstrated that almost half of unselected estrogen receptor alpha (ER-a) negative patients are estrogen receptor beta (ER-b) positive, and that these patients may benefit from adjuvant hormonal therapy[1]. African-American (AA) or Black patients are less likely to be ER-a positive and ER-b status is unknown.
**Purpose:** To evaluate and compare ER-b expression in unselected AA and non-AA patients, and determine relationships with ER-a, progesterone receptor (PR), and HER-2/neu (Her2) expression.
**Methods:** Immunohistochemical study using anti-ER-b antibodies (Affinity BioReagents). More than 10% staining was considered positive for ER-b. ER-a, PR and Her2 were determined by routine immunohistochemistry.
**Results:** 24 patients were studied with a mean age of 55 years old (range 34 to 71). There were 15 AA and 9 non-AA (predominantly white) patients. For the entire group, results were as follows; 38% ER-a positive (>5% staining), 33% PR positive (>5% staining), 38% Her2 positive (2 or 3+ staining) and 67% ER-b positive. 44% of ER-a positive and 80% of ER-a negative patients were ER-b positive; 38% of PR positive and 81% of PR negative patients were ER-b positive; 56% of Her2 positive and 73% of Her2 negative patients were ER-b positive. For AA patients results were; 33% ER-a positive, 27% PR positive, 27% Her2 positive and 73% ER-b positive. 60% ER-a positive and 80% ER-a negative patients were ER-b positive. 82% PR negative and 91% Her2 negative patients were ER-b positive. For non-AA patients, results were; 44% ER-a positive, 44% PR positive, 55% Her2 positive and 55% ER-b positive. 50% ER-a positive and 60% ER-a negative patients were ER-b positive. 25% PR negative and 50% Her2 negative patients were ER-b positive.
**Conclusions:** Levels of expression of ER-b were higher for AA or Black patients. For both AA and non-AA patients, ER-b expression was higher when ER-a status was negative. Black ER-a negative, PR negative and Her2 negative patients were most commonly ER-b positive.

1. Mann S. *et al*. Human Pathology 2001: Volume 32: 113-118.

**P21**

**HER-2/NEU OVEREXPRESSION IN BLACK WOMEN WITH BREAST CANCER IS NOT ASSOCIATED WITH NEGATIVE ER STATUS OR HIGHER STAGE DISEASE**

*Keith A. Dookeran, Elona Sciupokiene, Todd Frush, Houman Esmailzadeh, Frank Shin, Wendy Rogowski, Hung Tran, Jie Yang, Marin Sekosan[1], Rose Catchatourian[2], & Howard A. Zaren**.**
Departments of Surgery & Pathology[1], and Division of Medical Oncology[2], Cook County Hospital, 1835 West Harrison Street, Chicago, IL 60612 USA.

**Introduction:** HER-2/neu (Her2) overexpression in breast cancer is associated with worse prognosis, estrogen receptor (ER) negative tumors and more aggressive disease. African-American (AA) or Black women have poorer prognosis, are more often found to have ER negative tumors and present with higher stage disease[1].
**Purpose:** To compare Her2 overexpression in AA and non-AA patients and determine whether such expression is related to negative ER status, node positive status and higher disease stage at presentation.
**Methods:** 158 consecutive cases (106 AA and 52 non-AA) with breast cancer were evaluated for ER and Her2 expression with immunohistochemistry, and analyzed by race, stage (AJCC I vs. II-IV), and ER & node status.
**Results:** There were no significant differences between groups and 58% AA and 54% non-AA (predominantly white) patients were ER positive (> 5% staining). Only 28% AA and 29% non-AA of ER positive patients were Her2 positive (2 or 3+ staining). For ER negative patients, non-AA was almost twice as likely to be Her2 positive (54%), compared with AA (30%) (Chi-square p=0.05). Non-AA Her2 positive patients were more likely to have higher stage (II-IV) disease (46 vs. 30%). All (100%) non-AA Her2 positive ER negative patients were higher stage, in contrast to 64% of similar AA patients. The majority (64%) of AA Her2 positive ER negative patients were node negative. In contrast, most (67%) non-AA Her2 positive ER negative patients were node positive.
**Conclusions:** Her2 expression appeared to be similar for AA or Black patients, whether ER positive or negative, and was generally lower than expression for Non-AA patients. Her2 expression in AA patients was not associated with negative ER status, node positive status, or higher stage disease.

1. Joslyn S.A. & West M.M. Cancer 2000: Volume 88: 114-23.

© 2002 Cancer Research UK

**P22**
**A CHECKLIST FOR PATIENTS ON ENDOCRINE THERAPY (C-PET): RESULTS OF THREE YEARS USE AT A SINGLE INSTITUTION.**
RCF Leonard [1*], K M Malinovszky [2], D Cameron[1], S Douglas [1], M Dixon[1], C Love[1].
[1]Breast Unit, Western General, Edinburgh, [2] South West Wales Cancer Institute, Singleton Hospital, Swansea.

**Introduction:** Although hormonal therapies are considered to be relatively well tolerated, there is audit evidence that medical professionals underestimate the distress caused by certain symptoms[1]. Patients on chronic endocrine therapy may be given less time at clinics to discuss 'minor' problems compared to patients receiving more toxic and complex cytotoxic regimens. **Method:** Over a 3 year period, breast cancer patients receiving endocrine therapy, attending the Edinburgh Breast Unit, were asked to complete a checklist of the symptoms they were experiencing (C-PET). This single page checklist took no more than two minutes to complete and was taken into the consultation to be used as a prompt for doctors and nurses, and to provide a better insight into side-effects experienced and how they were affecting the patient. **Results**: In the Edinburgh Unit 748 patients completed forms. Data on type of endocrine therapy and the symptoms experienced by each patient were examined. Most patients were on tamoxifen (524), anastrozole (103) and megestrol acetate (35). Data for these three therapies are reported. Other therapies included letrozole, formestane, goserelin and various combinations of these. The proportion of patients experiencing specific symptoms is shown in table 1. Tamoxifen symptoms were similar in the metastatic and adjuvant treatment patients so the data are aggregated, although perhaps unsurprisingly breathlessness and low energy were reported more frequently in the metastatic group.

**Table 1. Percentage of patients reporting symptoms**

| | HF | SW | WG | NS | FR | SOB | PVB | PVD | DR | IR | EN |
|---|---|---|---|---|---|---|---|---|---|---|---|
| Tamoxifen | 58 | | 42 | 10 | 25 | 19 | 8 | 30 | 16 | 11 | 28 |
| Anastrazole | 51 | 51 | 30 | 28 | 21 | 32 | 4 | 13 | 10 | 17 | 48 |
| Megestrol acetate | 40 | 43 | 57 | 11 | 40 | 49 | 14 | 11 | 11 | 20 | 49 |

**KEY:** HF-hot flushes, SW-sweats, WG-weight gain, NS-nausea, FR-fluid retention, SOB-breathlessness, PVB-vaginal bleeding, PVD-vaginal discharge, DR-vaginal dryness, IR-irritability, EN-low energy.
Other symptoms mentioned by patients included joint pain/stiffness (anastrozole), hair loss, dizziness and visual disturbance.
**Conclusion:** This method was found to be a simple and speedy means of gaining information on symptoms associated with endocrine therapies, both in the clinic and as aggregated auditable data, and the data suggest that toxicity from endocrine therapies is more prevalent than previously reported

[1]Leonard RC& Lee L 1996 The Breast (5) 259-264.

**P23**
**DETECTION AND QUANTITATIVE MONITORING OF MINIMAL RESIDUAL DISEASE (MRD) IN ACUTE LYMPHOBLASTIC LEUKAEMIA (ALL) PATIENTS.**
Quinn F[1*], Crampe. M.[1], Browne P[1], O'Marcaigh A[2], Stallings R[2], McCann SR[1], Smith OP[1,2], O'Meara A[2], Breathnach F.[2] M. Lawler[1]. Depts. of [1]Haematology/Oncology St. James Hospital Trinity College Dublin Ireland; [2]Our Lady's Hospital for Sick Children, Crumlin, Dublin Ireland.

Minimal residual disease (MRD) detection is becoming an integral part of therapeutic protocols for childhood acute lymphoblastic leukaemia (ALL). Assessment for MRD at defined timepoints after diagnosis may allow risk stratification, prediction of relapse and the option of appropriately tailoring subsequent therapy. A number of protocols now utilise MRD results in the stratification of patients in clinical protocols. We have initiated a prospective study of MRD using both PCR based methodologies and multi-parameter flow cytometry in children entering into the MRC ALL97/99 (revised) trial. Samples (bone marrow) are collected from the patient at diagnosis and at days 8 and 28 during induction, at weeks 17 and 24 during intensification, at week 39 at the start of maintenance and thereafter at the end of treatment and at time of relapse if applicable. PCR protocols have been optimised to detect the five major translocations in childhood ALL: t(4;11) MLL-AF4; t(12;21) TEL-AML1; t(9;22) BCR-ABL p190 and p210; and t(1;19) E2A-PBX1. Sensitivity of detection ranges from $10^{-2}$-$10^{-3}$ with standard PCR to $10^{-4}$-$10^{-5}$ when using nested PCR with primers designed internally to the amplicon produced by standard PCR. At diagnosis, cells were examined by cytogenetics, flow cytometry and by routine qualitative RT-PCR for evidence of aberrant chromosome rearrangements or a leukaemia associated phenotype (LAP). Levels of MRD are monitored in translocation positive patients during treatment using real-time RT-PCR with 5' nuclease (Taqman) probes in accordance with the Europe Against Cancer/Biomed group. Sensitivity of detection is 1 aberrant mRNA transcript within a population of $10^{5}$-$10^{6}$ cells by PCR or . Patients who do not test positive for a chromosomal translocation are screened for clonal IgH, IgK, TCRδ and TCRγ(B cell ALL) or TCRδ, TCRγ, SIL-TAL (T cell ALL). Following heteroduplex analysis, major clonal bands are sequenced and allelic-specific primers designed and utilised with consensus 5' nuclease probes in real time PCR analysis. In parallel, the use of multiparameter flow cytometry to track MRD after initial therapy requires the detailed characterisation of an aberrant leukaemia associated phenotype (LAP) for each patient at diagnosis. Multiple combinations of antibodies from a panel of over 15 are used to test each diagnostic bone marrow sample for simultaneous expression of antigens. Patient-specific antibody combinations are then used in follow-up samples to quantitate MRD. A sensitivity to $10^{-4}$ has been achieved with this technique. To date, 30 patients have been enrolled in the study with a sampling range of 1-54 weeks. Minimal residual disease has been assessable to date in 23 patients using molecular and immunophenotypic analysis. MRD positivity has been detectable in 17/23 patients at Day 28 and in 14 patients MRD positivity has decreased over time. Evidence of MRD negativity has been detected in 5 patients, but follow up time is short. Continued prospective monitoring of MRD in childhood ALL will help stratify patients for further therapeutic intervention. Supported by the Childrens Leukemia Research Project.

**P24**
**MANAGEMENT OF ELDERLY PATIENTS WITH 'AGGRESSIVE' NON-HODGKIN'S LYMPHOMA (NHL) PRESENTING TO A SINGLE CENTRE OVER THREE DECADES**
SJ Strauss*[1], KW Last[1], AJ Davies[1], VK Cornelius[4], A Wilson[1], J Amess[2], A Norton[3], AZS Rohatiner[1], TA Lister[1], ICRF Medical Oncology Unit[1], Dept of Haematology[2], Dept of Pathology[3], St Bartholomew's Hospital, London, ICRF Statistics Group[4], Oxford, UK.

Between 1972 and 1998, 101 consecutive adults over the age of 70 years were referred to the Department of Medical Oncology, for the management of 'aggressive' NHL. Data were available for 96 patients, (56 women) with the following histological subtypes (Kiel classification): centroblastic (36), high grade unclassified (25), immunoblastic (22), 'other' (13). All patients underwent routine staging with clinical examination, CT scanning, bone marrow biopsy and routine haematology and biochemistry investigations, resulting in: 28 patients with stage I disease, 22 stage II, 14 stage III and 31 with stage IV disease. The median age was 77 years (range 70-93). 88 patients were treated with curative intent and 8 managed supportively. 66 patients received combination chemotherapy according to protocols used at the time, and 22 were treated with radiotherapy (RT) with selection based on clinical findings. No patients received growth factor support. 7 patients have been lost to follow up.
Nine patients are alive, with a median follow up of 5 years (range 0.6 to 11.4 years). The actuarial proportion predicted alive at 5 and 10 years is 31 % and 16%, respectively. 79 patients have died; 41 of lymphoma, 17 of treatment-related causes, 5 of other malignancies, 7 of other known co-morbidity, and 11 of unknown causes. 21 of 22 patients receiving RT completed planned therapy. In contrast, only 7 of 66 (12%) patients receiving chemotherapy completed the intended treatment at full dose; 15 did not to due to treatment-related death, 11 due to progressive disease, and 33 due to debility at diagnosis or resulting from treatment. Despite this, 22 patients (33%) achieved a complete response (CR) with an overall response rate of 62%. Early stage disease was a strong predictor of treatment response, with an overall response rate in stage I/II of 78% compared to that in stage III/IV of 41% ($p<0.001$). Age examined as a continuous variable did not predict for response to treatment. In the group that responded to treatment (CR and partial response) 5 year progression free survival (PFS) was 33%. Recurrence continued after 5 years, and at 10 years the PFS was 6%.

 © 2002 Cancer Research UK

These long-term results emphasise the importance of careful selection of therapy for this patient population and confirm that it is possible to achieve long survival for a small proportion of elderly people with 'aggressive' NHL. Advances in supportive care and the availability of additional but less toxic therapy may improve outcome.

**P25**

**THE SCHEDULE-DEPENDENT CYTOTOXICITY OF ETOPOSIDE IN LEUKAEMIC CELL LINES.**

WM Liu* and SP Joel.
Dept Medical Oncology, St Bartholomew's Hospital, London.

*Purpose:* Etoposide is a commonly used anti-cancer agent that exerts its cytotoxic effects by stabilising transient topoisomerase II/DNA complexes that ultimately result in cell death. As part of our studies, the *in vitro* activity of etoposide (0-0.4 μM) in a panel of leukaemic cell lines has been studied. *Method:* CEM and HL60 cells were cultured with etoposide following drug-schedules of equal exposure duration (ED: duration x concentration), and cell parameters, including cell cycle distribution, were assessed daily. *Results:* Proliferation assays showed etoposide to be an effective cytotoxic agent ($IC_{50}$ on day 5 of 0.39 μM and 0.15 μM in CEM and HL60 respectively), showing concentration and duration-dependent activity. Flow cytometric analysis revealed etoposide to induce a block in the G2/M phase of the cell cycle which was transient, as continued exposure resulted in an emptying of cells from this phase (%G2/M in cells with 0.4 μM on days 1, 3 and 5 was 62.1 ± 2.1%, 40.3 ± 6.4% and 12.3 ± 1.2% respectively, vs. 26 ± 2.9% in control cells with no added etoposide). Importantly, this emptying was associated with an increase in apoptosis (%A in cells with 0.4 μM on days 1, 3 and 5 was 11.5 ± 1.8%, 37.5 ± 6.5% and 75.7 ± 1.0% respectively). The comparison of a range of 8-day schedules with equi-ED consistently revealed a greater loss of percentage viability in cells cultured in schedules of longer duration of exposure. As an example, total % cell kill in CEM cells induced by a continuous exposure to 0.05 μM etoposide was significantly greater than induced by either a 4-day exposure to 0.1 μM or a single-day exposure to 0.4 μM etoposide. Furthermore, the total numbers of cells on day 8 were significantly smaller in schedules of longer duration (Table).

| | 8-day schedule (equi-ED = 0.4 μM.days) | | | |
|---|---|---|---|---|
| | **0 x 8d** | **0.4 μM x 1d + 0 x 7d** | **0.1 μM x 4d + 0 x 4d** | **0.05 μM x 8d** |
| **Total %CK*** | 12.3 ± 3.8% | 42.3 ± 5.9% | 125.2 ± 5.4% | 193.4 ± 15.9% |
| **CC on day 8** | 46.4 ± 5.8 | 42.6 ± 4.7 | 13.6 ± 3.6** | 4.2 ± 0.6*** |

*CK = cell kill, CC = cell counts ($x10^5$ /ml). *p<0.001 when comparing CK in any two schedules. **p<0.001 vs. 0.4 μM x 1d; ***p<0.001 vs. 0.1 μM 4d.*

*Conclusions:* In addition to confirming the importance of total exposure to etoposide in determining cytotoxicity, we have also highlighted the importance of schedule in determining drug efficacy. Studies are continuing to elucidate the mechanisms of this schedule effect.

**P26**

**SUBSEQUENT PRIMARY CANCERS AFTER TREATMENT FOR HODGKIN'S DISEASE AND NON-HODGKIN'S LYMPHOMA**

AFC Okines[1*], CR Radstone[2], C Thomson[2], CE Hayward[2], H Hancock[2], BW Hancock[2]
1 University of Sheffield, School of Medicine. 2. YCR Academic Unit of Clinical Oncology, Weston Park Hospital, Sheffield.

*Background:* Numerous studies have shown that patients treated for Hodgkin's Disease or Non-Hodgkin's Lymphoma are at an increased risk of developing both leukaemia and solid cancers.
*Aims:* To determine the incidence of subsequent primary malignancies in patients treated for lymphoma at Weston Park Hospital, Sheffield.
*Methods:* A cohort of 3913 consecutive patients (2131 male, 1783 female), diagnosed with Hodgkin's Disease or Non-Hodgkin's Lymphoma before July 2001, was identified using the Weston Park Hospital database. Follow up was 95.2% complete for these patients. A search was undertaken for all patients diagnosed with a subsequent primary malignancy. Four patients who received treatment for their lymphoma at another centre and 13 patients in whom a second malignancy was diagnosed within six months of lymphoma diagnosis were excluded.
*Results:* 63 new cancers in 60 patients were identified. The distribution of subsequent malignancies by lymphoma diagnosis and sex are expressed in the table below.

| Type of Subsequent Primary | HD Male | HD female | NHL Male | NHL Female | Totals |
|---|---|---|---|---|---|
| Breast Carcinoma | 0 | 8 | 0 | 4 | 12 |
| Lung Carcinoma | 2 | 2 | 6 | 2 | 12 |
| Leukaemia or Myelodysplasia | 4 | 2 | 2 | 0 | 8 |
| Colorectal Carcinoma | 1 | 0 | 4 | 3 | 8 |
| Others | 9 | 3 | 9 | 2 | 23 |
| Total | 16 | 15 | 21 | 11 | 63 |

At the close of the study, seven patients (11.67%) were alive, 52 patients (86.67%) had died and one (1.67%) had been lost to follow up. Registered cause of death included the subsequent malignancy in 49/52 patients (94.23%) Duration of follow-up ranged from six to 385 completed months (mean 115.8 months).
*Conclusions:* This study confirms a significant risk of subsequent primary malignancies in patients treated for lymphoma. Actual relative risk and the relationship to different treatment modalities need to be evaluated in this population.

**P27**

**A RETROSPECTIVE STUDY OF PATIENTS WITH PAROTID LYMPHOMA AT THE PRINCESS MARGARET HOSPITAL, TORONTO.**

Rebecca Herbertson*
Clinical Fellow in Haemato-oncology, St Bartholemew's Hospital, London

The aim of this study was to gain an understanding of the natural history of parotid lymphoma and how it has been treated over the last 30 years at The Princess Margaret Hospital in Toronto. It was intended to identify patient and tumour characteristics, treatment received and trends in response and outcome. 47 patients were identified as having biopsy proven primary parotid lymphoma between 1967 and 1998. Data was collected from the hospital database, the patient notes and the hospital computerised records. The endpoints evaluated were disease free survival and overall survival. The median age at diagnosis was 63yrs. Nine patients had documented bilateral disease (6 of these had MALT lymphoma) and three patients (6.4%) had B-symptoms at presentation. The Karnofsky performance status ranged from 30-100%, the median being 90%. Fourteen patients had high-grade lymphoma (12 of these were diffuse large cell) and 33 had low-grade disease (13 of these were MALT lymphoma). The majority of patients presented with an asymptomatic lump (91.5%). At diagnosis 43 patients had stage 1 and 2 disease, and 4 patients had stage3 and 4 disease. Only 3 patients reported B symptoms and 19 patients had a tumour bulk of greater that 5 cms. Of the 13 patients with MALT lymphoma 5 patients were known to have a pre-existing diagnosis of Sjogrens disease. Three patients received bilateral, and ten patients received unilateral radiotherapy alone. Two received chemotherapy alone, and 2 received surgery alone. Fourteen patients received surgery followed by radiotherapy to the effected parotid gland. Two patients received surgery followed by bilateral parotid radiotherapy. Five patients had initial surgery, unilateral radiotherapy and then systemic chemotherapy. A further 7 patients studied underwent unilateral radiotherapy followed by chemotherapy. Following first line therapy, thirty-six patients achieved a complete remission. Two patients had progressive disease despite treatment and one demonstrated a partial remission. Seventeen out of 47 patients were later documented to have relapsed and five of these had evidence of more than one relapse. Of those who relapsed 2 patients had follicular mixed cell, 4 had follicular large cell, 4 had small cell, 4 had diffuse large cell, 1 had diffuse mixed and 2 had MALT lymphoma. Disease free survival and overall

© 2002 Cancer Research UK

survival were calculated for each histological type of lymphoma. The patients with diffuse large cell and T cell lymphoma had the shortest DFS and OS (33(mean) and 50 months respectively), and those with follicular mixed cell lymphoma had the longest mean DFS and OS (99 and 152 months respectively). This study looked at a sample of patients with different types of primary lymphoma of the parotid gland and demonstrated that in general it is a localised disease with an indolent course. The treatment received and subsequent outcome of disease was dependent on the histological type and stage at diagnosis. Because MALT lymphoma was recognised as a disease entity in 1994, the number of patients diagnosed with this (and their long-term outcome) is likely to have been underestimated in this sample. A further study to review all the histological diagnoses on this group of patients is planned for the future.

**P28**

**THE SYSTEMIC INFLAMMATORY RESPONSE PREDICTS BOTH CANCER SPECIFIC AND NON-CANCER SURVIVAL IN PATIENTS WITH MALIGNANT LYMPHOMA**

Maqsood M Elahi[1], Donald C McMillan[1], Colin S McArdle[1], Wilson J Angerson[1], Jennie Johnstone[2], Naveed Sattar[2]. 1. University Department of Surgery, Royal Infirmary, Glasgow G31 2ER, UK.
2. University Department of Biochemistry, Royal Infirmary, Glasgow G31 2ER, UK.

**Background**: The assessment of prognosis in patients with advanced lymphoma is problematical. The value of C-reactive protein concentration in this context has not been clearly defined.
**Methods**: Patients with a diagnosis of lymphoma (Hodgkins n=114 and non-Hodgkins lymphoma n=40) and who had measurements of C-reactive protein and albumin were studied. **Results**: Median survival, from the time of sampling was 241 days. On univariate analysis there was a significant relationship between C-reactive protein, albumin concentrations and the duration of cancer specific survival ($p<0.0001$). In contrast, tumour type ($p<0.05$) and C-reactive protein ($p<0.0001$) were related to the duration of non-cancer survival. The hazard ratio associated with a unit increase in $\log_{10}$ C-reactive protein concentration (i.e. a tenfold increase in C-reactive protein concentration) was 74.01 (95% CI 30.07- 182.16) and 4.622 (95% CI 2.325-9.190) for cancer specific and non-cancer survival respectively.
**Conclusions**: The results of the present study demonstrate that patients with advanced lymphoma have evidence of a systemic inflammatory response and the magnitude of that response strongly predicts the duration of cancer specific and non-cancer survival.
Keywords; C-reactive protein, albumin, lymphoma, cancer specific survival.

**P29**

**CYTOKERATIN 19 IMMUNOHISTOCHEMISTRY AND ASSESSMENT OF DEPTH OF MYOMETRIAL INVASION IN STAGE I ENDOMETRIAL CANCER**

Alexander-Sefre F, Singh N, Jacobs I., Gynaecological Cancer Research Unit, St Bartholomew's Hospital, London

**Introduction:**
Approximately 50% of endometrial cancer (EC) deaths occur in patients with stage I disease at presentation. Depth of myometrial invasion is the most important risk factor for lymph node metastasis and mortality. Assessment of depth of invasion is currently done by light microscopic examination of H&E stained sections.
Tumour cells can evade detection by light microscopy if they are isolated and vastly outnumbered by myometrial and inflammatory cells. In non-gynaecological cancers, immunohistochemical staining of tumour cells has proven value in detecting isolated infiltrating cells and lymphovascular invasion (LVI), which correlate well with tumour recurrence.
Cytokeratin 19 belongs to a family of 20 related polypeptides that are constituents of the intermediate filaments of epithelial cells. This protein is expressed in normal and neoplastic endometrial epithelial cells.
**Objective:**
To investigate the role of CK-19 immunohistochemistry (IHC) in assessing myometrial invasion in stage I EC and to determine its clinical importance.
**Methods:**
A total of 111 stage I EC patients with minimum 4 years follow-up were selected. Representative H&E stained sections were re-examined by a pathologist to identify blocks containing tumour with maximum myometrial invasion. Four micron sections from the selected blocks were stained for CK-19 using a standard avidin-biotin method. The immunostained sections were then examined by the same pathologist and the results compared with H&E findings. Cases suspected to have LVI were dually stained with CD-31 and Pancytokeratin to confirm true LVI.
**Results:**
Endometrial tumour cells showed positive though variable immunoreactivity for CK-19. Immunohistochemical assessment revealed deeper myometrial invasion than detected on H&E staining in seven cases (6 %). Thirty four cases were noted to have LVI that was not detected on H&E sections. CD-31 staining confirmed true LVI in 29 cases (26%).
**Conclusions:**
CK-19 IHC can detect isolated infiltrating tumour cells. It therefore has potential advantage over routine histology in accurate assessment of myometrial invasion in stage I EC. CK-19 IHC identifies lymphovascular invasion with much greater sensitivity than H&E staining by CK-19 IHC.
We aim to correlate the presence of isolated infiltrating cells and LVI identified solely by CK-19 IHC with known adverse clinico-pathological features, disease recurrence and overall survival.

**P30**

**RANDOMISED PHASE TWO STUDY OF CARBOPLATIN VERSUS CARBOPLATIN AND THALIDOMIDE IN PATIENTS WITH OVARIAN CANCER, WITH EVALUATION OF POTENTIAL SURROGATE MARKERS OF ANGIOGENESIS.**

*J.P. Braybrooke[1], S. Madhusudan[1], A. Blann[3], S. Wilner[1], E. Flanagan[1], A. Jenkins[2], C. Echeta[1], T.Perren[2], T.S. Ganesan[1]
Cancer Research Medical Oncology Units, Churchill Hospital, Oxford, UK[1] and St. James' Hospital Leeds, UK[2]; Haemostasis, Thrombosis and Vascular Biology Laboratory, University Department of Medicine, City Hospital, Birmingham, UK[3]

**Background**: Thalidomide is anti-angiogenic and has activity as a single agent against several tumour types. The mechanism of action of thalidomide has not been well characterised and its safety in combination with chemotherapy not assessed.
**Methods:** Patients receiving carboplatin (AUC 7) every 4 weeks for stage Ic – IV ovarian cancer were randomised to 6 months of thalidomide 100mg daily or no additional treatment. Surrogate markers of angiogenesis, including vascular endothelial growth factor (VEGF), basic fibroblast growth factor (bFGF), von Willebrands factor (vWF), vascular cell adhesion molecule-1 (VCAM-1) and E-selectin were measured before treatment and after each cycle of chemotherapy. Patients randomised to thalidomide had sensory nerve action potentials (SNAP) performed every 2 months.
**Results:** 34 patients (Median age 64 years, range 41 - 88 years) have been recruited (16 carboplatin, 18 carboplatin and thalidomide) and 26 are evaluable to date. There were no significant differences in response rates or toxicity between the groups. No patients on thalidomide had a significant fall in SNAP. Preliminary analysis (n=21) showed elevated baseline serum VCAM-1 and urinary VEGF in patients with more than 2cm residual disease post-operatively compared to those with less than 2cm. There was a significant decrease (paired T-test, $p < 0.05$,) in E-selectin and VCAM-1 on paired samples, between baseline and end of treatment for all patients analysed together. There were no significant differences between the two patient groups for any of the markers analysed.
**Conclusions**: Low dose oral thalidomide can be safely administered in combination with carboplatin chemotherapy. Potential surrogate markers of angiogenesis fall in response to both carboplatin alone and carboplatin plus thalidomide.

 © 2002 Cancer Research UK

### P31

### COX-1 AND COX-2 EXPRESSION IN INVASIVE CERVICAL CANCER: RELATIONSHIP TO PROGNOSIS AND CLINICOPATHOLOGICAL FACTORS

Athavale RD**, Clooney K[#], O'Hagan J*, Shawki H[#], Clark AH[#], Green JA*
**Liverpool Women's Hospital *University of Liverpool [#] Arrowe Park Hospital

**BACKGROUND**: COX-1 and COX-2 are members of the cyclooxygenase enzyme family, which may have a role in tumour invasion and influence apoptosis. COX-2 was significantly overexpressed in women treated surgically for early stage cervical carcinoma (FIGO stage IB) with lymph node or parametrial involvement (Ryu et al . Gynecol Oncol 2000;76:320-25). COX-2 expression has also been linked with reduced cause-specific survival and pelvic control in women treated with radiotherapy (Gaffney et al. Am J Clin Oncol 2001 Oct;24(5):443-6). The purpose of our study was to assess the role of COX-1 and COX-2 expression in the prediction of survival in early stage cervical carcinoma and to examine the relationship between the expression and clinicopathological factors. **PATIENTS AND METHODS**: Women diagnosed to have early stage cervical carcinoma and treated at the same cancer centre between 1981 and 1990 and for whom tissue blocks were available were included in this study. The median followup interval was 157 months (60-212 months). 27/28 women had squamous cell carcinoma and one had cervical adenocarcinoma. 23/28 tumours were moderate to poorly differentiated and 2/28 were well differentiated. The treatment was with surgery alone (n=17), radiotherapy alone (n=3), surgery and radiotherapy (n=3) and surgery with radiotherapy and subsequent chemotherapy (n=5). COX-1 and COX-2 expression were assessed by immunohistochemistry (IHC) by the same pathologist (AC) who was blinded to the survival outcomes. A four point score (0-4% or negative = 0, 5-24%=1, 25-49%=2, 50-74%=3 and 75-100%=4) was allocated for distribution and intensity was recorded on a 3 point score. Survival curves were plotted for distribution alone and using a combined score of distribution and intensity (0-3=low, 4-7=high). Prognostic factors examined included histological grade, tumour size at initial surgery and lymph node spread of tumour. The data was analyzed with SPSS release 10.0. **RESULTS**: COX-1and COX-2 were expressed in 61%(17/28) and 57% (16/28) of tumours respectively. 7 out of 8 women who died (between 4-33 months) overexpressed COX-1 (p=0.04) and 5 out of 8 expressed COX-2. Women with a weak expression of COX-2 (5-24%) and those with a low combined score for COX-1 showed a trend towards an improved survival as compared to strong expressers and high combined scores respectively. Moderate to poorly differentiated tumours appeared to overexpress both COX-1 (16/23 versus 17/28 overall) and COX-2 (15/23 versus 16/28 overall). There was no significant difference between tumour size and COX-1 or COX-2 expression. 2 out of 3 women with lymph node spread expressed both COX-1 and COX-2. **CONCLUSION**: This study suggests that increased expression of COX-1 and COX-2 may be associated with a poorer survival prognosis. A prospective evaluation of the cyclooxygenase enzymes particularly COX-2 may prove to be useful in selecting women with early stage cervical cancer for chemoradiotherapy.

### P32

### p53, pRB AND EGFr EXPRESSION IN RELATION TO (HPV) SUB-TYPE IN THE TRANSFORMATION OF CERVICAL INTRAEPITHELIAL NEOPLASIA (CIN).

McLeish R[1], Southern S[2], *Green JA[3], MacDicken IA[2]. [1]Department of Surgery, Royal Liverpool University Hospital, Liverpool L7 8XP, [2]Department of Pathology, Royal Liverpool University Hospital, Liverpool L7 8XP, [3]Clatterbridge Centre for Oncology, Bebington Merseyside CH63 4JY

The aim of this study was to assess the interrelationships between growth regulatory genes, human papillomavirus (HPV) and grade of CIN. The frequency of HPV-DNA in this retrospective series of 200 cases was 72% of cases assessed by a sequential combination of NISH and PCR based analysis. A strong statistical association between the presence of "high risk" oncogenic HPV types (16/18 and 31/33/51) and the development of CIN2 or 3 was observed ($X^2$=12.49:p<0.0005). Similarly, the "low risk" non-oncogenic HPV types (6/11) were associated with the development of low-grade CIN lesions (CIN1 or wart virus). This data supports the hypothesis that the transforming capacity of HPV is subtype specific. The expression of p53 was reduced in oncogenic HPV type tissues as compared to non-oncogenic sub-types (©$^2$=30.8 :P<=0.0003), and was also less in higher grade CIN lesions as compared to CIN1 (©$^2$=5.5: p<0.02). This loss of expression supports the hypothesis that p53 is functionally inactivated by complex formation with the E6 and E7 oncoproteins of "high risk" HPV types. A similar association was found between loss of RB expression and increasing grade of CIN (©$^2$=16.9: p<0.0001). An increased level of p53 was observed in the parabasal layer of dysplastic lesions with non-oncogenic HPV sub-types and deemed to be persistent wild type gene products. EGFr expression was found to be reduced in expression with decreased p53 expression (©$^2$=6.07:p=0.014), but there was no relation to the other variables.
These results suggest that the presence of oncogenic HPV types and the loss of p53 and pRB expression may be prognostic indicators in CIN, and may complement histopathological assessment of the malignant potential of dysplastic lesions.

### P33

### GRANULOSA CELL AND SERTOLI-LEYDIG CELL OVARIAN TUMOURS – A 10 YEAR REVIEW OF CASES AT CLATTERBRIDGE CENTRE FOR ONCOLOGY

*Moss EL[1], Steele PRM[2], Davies-Humphries J[1], Green JA[3]
[1]Department of Gynaecology, Countess of Chester Hospital, Chester CH2 1UL. [2]Department of Pathology, Countess of Chester Hospital, [3]Clatterbridge Centre for Oncology, Bebington, Merseyside CH63 4JY

**Introduction:** Granulosa cell and Sertoli-Leydig cell tumours account for 5-6%of all malignant ovarian cancers. Previous studies have shown that the majority of tumours are FIGO stage 1 at presentation and are associated with a 70-90% 5 year survival rate. The aim of this study was to look at the current patterns of presentation, treatment and outcome of these tumours.
**Methods:** A 10 year retrospective notes review. Information was abstracted on patient characteristics, disease presentation and stage, treatment and outcome, including relapse and death. Histology reports were reviewed by a pathologist.
**Results:** A total of 15 granulosa cell tumours were recorded, with a mean age of 62.1 years (range 44-83 years) and documented stages Ia:n=1, Ib:n=2, Ic:n=5, II:n=2, III:n=4, unknown in 1 case. Surgery composed of TAH BSO in 11 cases, and the median tumour size was 12cm (2.5-23cm). Washings were positive in 6 cases. Additional treatment was pelvic radiotherapy in one case. Six patients received platinum based chemotherapy, of whom 3 have recurred at 8, 17 and 72 months. One patient had died at 12 years and 12 are alive at a median of 5 years. Eight Sertoli-Leydig tumours were seen, with a mean age of 47 years (range 17-63 years), and recorded stages I:n=2, II:n=2, III:n=2 and 1 stage IV, unknown in 1 case. Size ranged from 5.5-19cm. Five tumours ruptured at resection. TAH BSO was carried out in only 3 cases and unilateral oophorectomy in 5 cases. Six patients received platinum based chemotherapy, of whom 5 responded, but 4 have relapsed at 6-19months. Three patients are alive at 8-48 months, the remainder are dead at 6-30 months.
Histology reports were available on 18 patients and these showed a great variation in the extent and content of both the macroscopic and microscopic descriptions. Information which may have prognostic significance, including capsule penetration, appearance of the nuclei and mitotic counts, was often not recorded. Only 4 of the 18 cases were referred for an external expert opinion.
**Conclusions:** This study highlights the inconsistency in the diagnosis, management and treatment of women with granulosa cell or Sertoli-Leydig cell ovarian tumours. This study is now being extended to cover the Merseyside and Cheshire area, a population of over 2 million people, from 1980-2000, and will include an audit of the pathology reporting.
Significant improvement in the management of rare cases will only be obtained by studies with large patient numbers, which will only be possible through international collaboration. A central tumour registry with co-ordinated data collection and prospective clinical trials is proposed.

© 2002 Cancer Research UK

